# Fiber Fabry-Perot Force Sensor with Small Volume and High Performance for Assessing Fretting Damage of Steam Generator Tubes

**DOI:** 10.3390/s17122899

**Published:** 2017-12-13

**Authors:** Peijian Huang, Ning Wang, Junying Li, Yong Zhu, Jie Zhang

**Affiliations:** The Key Laboratory of Optoelectronic Technology & System (Ministry of Education), Chongqing University, Chongqing 400044, China; 20113269@cqu.edu.cn (P.H.); junyingli@cqu.edu.cn (J.L.); zhuyong@cqu.edu.cn (Y.Z.); zhangjie@cqu.edu.cn (J.Z.)

**Keywords:** fiber optics sensor, steam generator tube, fretting damage, collision force measurement

## Abstract

Measuring the radial collision force between the steam generator tube (SGT) and the tube support plate (TSP) is essential to assess the fretting damage of the SGT. In order to measure the radial collision force, a novel miniaturized force sensor based on fiber Fabry-Perot (F-P) was designed, and the principle and characteristics of the sensor were analyzed in detail. Then, the F-P force sensor was successfully fabricated and calibrated, and the overall dimensions of the encapsulated fiber F-P sensor were 17 mm × 5 mm × 3 mm (L × W × H). The sensor works well in humid, high pressure (10 MPa), high temperature (350 °C), and vibration (40 kHz) environments. Finally, the F-P force sensors were installed in a 1:1 steam generator test loop, and the radial collision force signals between the SGT and the TSP were obtained. The experiments indicated that the F-P sensor with small volume and high performance could help in assessing the fretting damage of the steam generator tubes.

## 1. Introduction

Steam generator tube rupture (SGTR) has been addressed as one of the most significant safety issues in past decades when operating nuclear power plants. According to an independent investigation of American pressurized water reactor (PWR) nuclear power plants, 75% of all failure risks in pressurized water reactor nuclear power plants are induced by SGTR-related faults [1]. The investigation results also show that the fretting damage due to the fluid motion and thermal energy transmission between the SGT and the tube support plate (TSP) is the most important cause of fatigue cracks in the SGT. The fretting work was used to assess the fretting damage of the SGT [2,3,4,5,6]. To calculate the fretting work, the radial impact force and the axial displacement between the SGT and the TSP need to be measured. The radial impact force is more difficult to measure than the axial displacement in the complex working environment of the SGT. Therefore, measuring the collision force between the SGT and the TSP is urgently needed to assess the fretting damage of the SGT, and thus to monitor the health condition of the SGT. 

**Figure 1:**
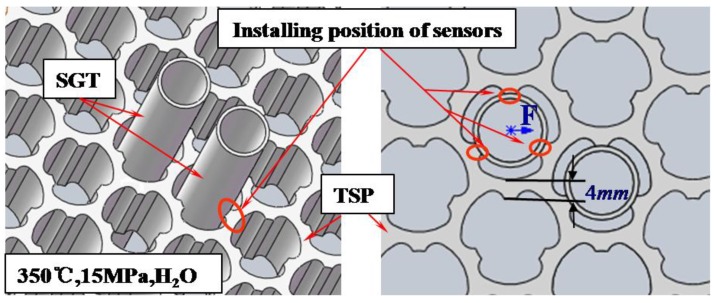
Schematic structure of steam generator tube (SGT) and tube support plate (TSP) and installing position of force sensor.

As shown in Figure 1, steam generator tubes are arranged closely in a high temperature and pressure environment (350 °C, 15 MPa, humid). Therefore, this working environment requires force sensors with large measurement range, small volume (especially, the height must be less than 4 mm), and good heat resistance, pressure resistance, and waterproofness. Meanwhile, since the impact time of radial collision force is short [6], a corresponding high-speed force sensor demodulation system is required. The first and urgent step to accessing the fretting damage of the SGT is to investigate the force sensors with high performance in a real steam generator tube. 

Existing electric force sensors are too large to be installed in the narrow space at the contact position between the SGT and the TSP. Compared with the electric force sensors, the fiber sensors have outstanding advantages due to their small size and anti-electromagnetic interference [7]. Mo et al. reported a miniature and magnetic-resonance-compatible optical Fabry-Perot (F-P) force sensor [8], achieving a force sensing range of 0–5 N with a resolution of 0.1 N. Noh et al. developed a three-axis fiber-optic body force sensor structure for flexible manipulators with a measurement range of 0–21 N, being suitable for inclusion in instruments and robotic tools for minimally invasive surgery [9]. Tang et al. reported a sensitive force sensor using two identical fiber Bragg gratings (FBGs), achieving a sensitivity of (1.16 ± 0.06) × 10^3^ dB/N [10]. Zychowicz presented a fiber-optic force sensor, operating in the configuration of a birefringent fiber based on a Sagnac interferometer, with a measurement range of 0–2 N [11]. Tsuda et al. developed a small-diameter FBG sensor to detect the impact damage of carbon fiber reinforced plastic [12]. These sensors are based on fiber F-P or fiber Bragg gratings, but they are too large for the small gap at the contact position between the SGT and the TSP. Further, these sensors are insufficient to meet the range requirements (range of 0–200 N, resolution of 1 N) in the SGT fretting damage assessment applications. 

In this paper, a novel micro-machined fiber F-P force sensor was developed. Its principle, characteristics, and structure parameters were analyzed in detail. Moreover, the sensors were fabricated and then tested in high-pressure, humid, high-temperature, and vibration environments. The sensor worked successfully on the 1:1 steam generator test loop (which simulated a real steam generator with the same geometry and system design). 

## 2. Design of F-P Force Sensor

The sensor has to meet two key requirements: first, the measurement range has to be around 200 N with the resolution of about 1 N; second, the size of sensors has to be small enough. Therefore, there are three key points to be considered during the designing process. (1) The structure of the force measuring element should be carefully designed to maintain the parallelism of the two surfaces of the F-P cavity. The beam length should be appropriately selected when the beam thickness and beam width are limited by the small installation space. (2) Considering the working conditions, the material with higher elasticity modulus, larger tensile strength, and lower heat expansion coefficient is preferable for the force measurement element. (3) To obtain the real-time F-P cavity length, the intensity output from the F-P cavity in the sensor is processed by non-scanning correlation demodulation method [13,14], so the resolution of the F-P force sensor depends on the minimum F-P cavity length variation that the demodulation system can distinguish. 

### 2.1. Structure Design and Analysis

**Figure 2:**
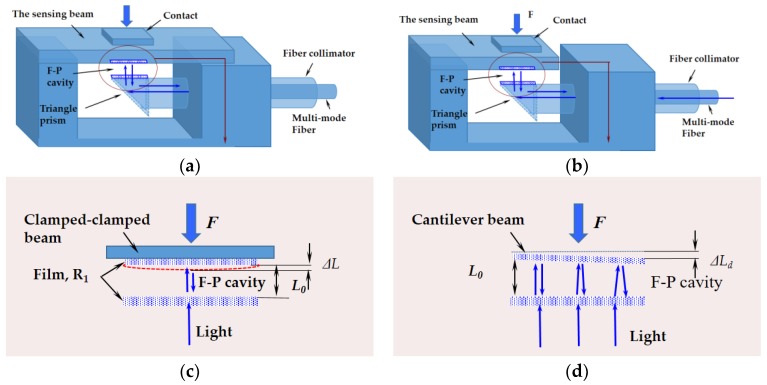
Three-dimensional structure of our Fabry-Perot (F-P) force sensors with (**a**) clamped-clamped beam element; (**b**) cantilever beam element; a simplified structure of the F-P cavity length change induced by the force F for (**c**) a clamped-clamped beam; (**d**) a cantilever beam.

Due to the small gap at the contact position (less than 4 mm) between the SGT and the TSP, a 45° reflection prism, a fiber collimator, and a sensing beam with suitable elastic force measuring element were utilized. The clamped-clamped beam and the cantilever beam are commonly used structures for a sensing beam, as shown in Figure 2a,b. The beam deflections of both clamped-clamped beam and cantilever beam are equal to the change of the F-P cavity length (see Figure 2c,d). 

The F-P cavity length changes when a force is exerted on the force-sensitive element (sensing beam). The cavity length variation is related to the magnitude of the applied force *F*. The non-scanning correlation demodulation method is used to obtain the F-P cavity length variation induced by the force *F*. The relationship between the F-P cavity length variation and the applied force *F* in two different sensing structures is given by:

(1) $$F_{1}=\Delta L\frac{16Ebh^{3}}{l^{3}}=\left(L_{0}-L_{0}{}^{\prime }\right)\frac{16Ebh^{3}}{l^{3}}$$

(2) $$F_{2}=\Delta L_{d}\frac{3Ebh^{3}}{l^{3}}=\left(L_{0}-L_{0}{}^{\prime }\right)\frac{3Ebh^{3}}{l^{3}}$$

where *F*_1_ is the force exerted on the clamped-clamped beam, *F*_2_ is the force exerted on the cantilever beam, *E* is the elastic modulus of the beam material, *l* is the beam length, *h* is the beam thickness, *b* is the beam width, *L*_0_ is the original F-P cavity length, *L*_0_′ is the F-P cavity length induced by the force, Δ*L* is the F-P cavity length variation, and *R*_1_ is the reflective index.

Based on Equations (1) and (2), the measurement range of the F-P force sensor is proportional to the maximum F-P cavity length variation Δ*L*_max_ that the demodulation system can demodulate; the measuring accuracy is related to the minimum F-P cavity length variation Δ*L*_min_ that the demodulation system can distinguish.

Compared with the clamped-clamped beam element, the cantilever beam element has higher sensitivity and larger measurement range. However, an exerted force would severely reduce the degree of parallelism between the two reflective surfaces of the F-P cavity in the sensor (see Figure 2d), while the parallelism must be strictly guaranteed in order to ensure the good optical performance of the F-P cavity. Therefore, a systematic analysis must be done to aid in the design of the sensor structure.

**Figure 3:**
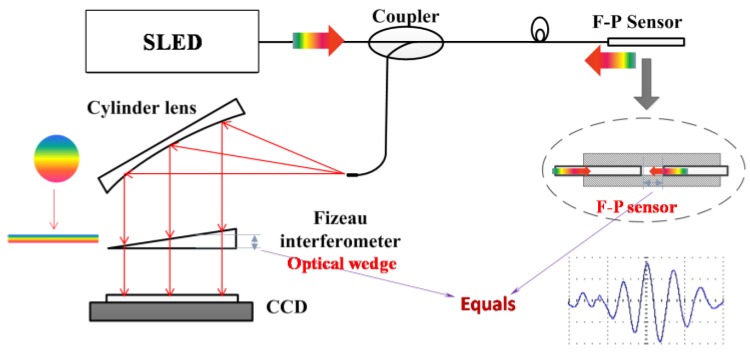
A schematic of the non-scanning correlation demodulation method. (SLED, super luminescent light emitting diode; CCD, charge coupled devices)

For the cantilever beam force sensor, *L* is no longer a fixed value with a large deformation of F-P cavity. The F-P cavity could be decomposed into infinite numbers of micro F-P cavities. The signal of the force sensor can be demodulated by non-scanning correlation demodulation method [13], shown in Figure 3. Additionally, the intensity on the charge coupled devices (CCD) could be calculated by integration with the integration step of Δ*L_step_*, expressed by

(3) $$I_{out}\left(x\right)=\frac{\Delta L_{step}}{\Delta L}\int_{\lambda_{\min}}^{\lambda_{\max}}\int_{L-\Delta L_{d}}^{L}\frac{2R_{1}\left(1+\cos\frac{4\pi L}{\lambda }\right)}{1+R_{1}^{2}+2R_{1}\cos\frac{4\pi L}{\lambda }}\cdot \frac{{\left(1-R_{2}\right)}^{2}}{1+R_{2}^{2}-2R_{2}\cos\left(\frac{4\pi x\tan\theta }{\lambda }\right)}e^{-\frac{{\left(\lambda -\lambda_{p}\right)}^{2}}{B_{\lambda }^{2}}}I_{0}dLd\lambda$$

where *R*_1_ is the reflectivity of the F-P cavity end face, *R*_2_ is the reflectivity of the Fizeau interferometer, *B_λ_* is the bandwidth of the super luminescent light emitting diode (SLED), *λ_p_* is the central wavelength of the SLED spectrum, *λ*_min_*~λ*_max_ is the wavelength range of SLED, *θ* is the optical wedge angle, *L* is the F-P cavity length, *x* is the optical wedge length equal to the photoreceptor length of the CCD, *I*_0_ is the input light spectrum density, and $\exp(-{\left(\lambda -\lambda_{p}\right)}^{2}/B_{\lambda }^{2})$ results from the Gaussian lineshape of the SLED spectrum.

**Figure 4:**
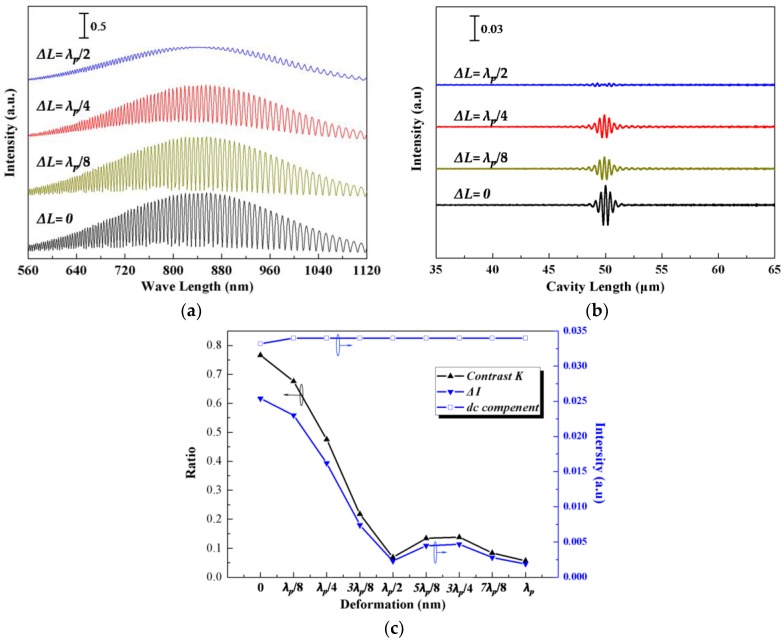
Simulated signals of fiber F-P sensor with different deformation: (**a**) F-P reflective light intensity; (**b**) demodulation signals; (**c**) contrast *K*, Δ*I*, *dc* component.

The signals of the F-P force sensor demodulated by the non-scanning correlation demodulation method could be discussed by numerical computation based on Equation (3), and the corresponding demodulated signals are shown in Figure 4a,b at different Δ*L* values (other parameters are *R*_1_ = 0.2, *R*_2_ = 0.5, *L* = 50 μm, *θ* = 0.05°, *λ_p_* = 840 nm, and *B_λ_* = 60 nm). Note that when the height of the optical wedge equals the cavity length of the F-P sensor, a maximum value of the corresponding intensity on the CCD pixel appears. The CCD pixel position where the maximum value appears is targeted to find the corresponding demodulated F-P cavity length. 

Three parameters are used to evaluate the demodulation quality: Δ*I* is the difference between *I*_max_ and *I*_min_, *dc* is the averaged value of *I_out_*, and the signal contrast *K* is the ratio of the F-P cavity length signal intensity (*I*_max_ − *I*_min_) and the dc component. The three parameters are calculated and plotted in Figure 4c. It indicates that when Δ*L* is less than *λ_p_*/4, it is easy to demodulate the signal. The contrast ratio is reduced with the increase of Δ*L*. When *ΔL* reaches *λ_p_*/2, the demodulated signal almost disappears. When Δ*L* is larger than *λ_p_*/2, it is more difficult to demodulate the signal. In order to weaken the incline effect of the sensing beam, the value of Δ*L* should be kept smaller than *λ_p_*/2. The above analyses indicate that the tilt deformation of the F-P cavity must be less than *λ_p_*/2 during the sensing, which limits the measurement range of the force sensor. Compared with a cantilever beam element, the clamped-clamped beam element performs better in maintaining the parallelism between the two reflective surfaces of the F-P cavity, which achieves a larger measurement range of the force sensor. Therefore, in this paper, the clamped-clamped beam element was used as the sensing beam in the subsequent discussion, fabrication, and experiments.

### 2.2. Material and Size Requirement

First, materials with high-temperature and high-pressure resistance were used to structure the F-P force sensor. In this design, optical fibers made of quartz (melting point at ~1750 °C) were used, and 3Cr13 (melting point at ~1371 °C) or 06Cr19Ni10 (melting point at ~1400 °C) stainless steel was used for the clamped-clamped beam. Second, in order to guarantee the rigidity of the supporting structure when exerting the maximum force of 200 N, stainless steel was used to package the F-P force sensor.

In order to optimize the dimension and materials used, three important clamped-clamped beam element parameters are discussed as follows:

(4) $$S=\frac{\Delta L}{F_{c}}=\frac{l^{3}}{16Ebh^{3}}$$

(5) $$F_{m}=\left|\sigma_{b}\right|\cdot \frac{4bh^{2}}{3l}$$

(6) $$f_{0}=\frac{22.37}{2\pi l^{2}}\sqrt{\frac{Eh}{12\rho }}$$

where *S* is the sensitivity of the sensor, *F_m_* is the permissible bending stress of the sensor, *f*_0_ is the natural frequency of the sensor, *F_c_* is the force exerted on the clamped-clamped beam element, *E* is the elasticity modulus of the element material, *l* is the beam length, *b* is the beam thickness, *h* is the beam width, Δ*L* is the F-P cavity length variation, *σ_b_* is the tensile strength of the element material, and *ρ* is the density of the element material.

The sensitivity *S* depends on the size and material of the sensing beam. The permissible bending stress *F_m_* has to be large enough to prevent the F-P sensor failure caused by the fracture of the sensing beam. The natural frequency of the F-P force sensor has to be far away from the frequency of the SGT radial collision force and the sampling frequency of demodulation system in order to avoid the error caused by the resonance. 

Based on Equations (4) and (6), *S*, *F_m_*, and *f*_0_ were calculated with different beam lengths *l* of 3 mm, 4 mm, and 5 mm (other parameters are *b* = 2 mm, *h* = 0.6 mm, *L* = 50 μm, Δ*L*_max_ = 25 μm, Δ*L*_min_ = 30 nm) respectively. The calculation results based on 06Cr19Ni10 stainless steel and 3Cr13 stainless steel are shown in Table 1.

The results show that the sensitivity *S* becomes larger and other parameters become smaller with the increase of the beam length. Since the permissible bending stress *F_m_* of 06Cr19Ni10 stainless steel is unable to meet the requirement of the measurement range, the 3Cr13 stainless steel was selected for the F-P force sensing system, with the sensing beam length of 4 mm. Therefore, the dimensions of the 3Cr13 stainless steel force sensing beam were designed to be *b* = 2 mm, *h* = 0.6 mm, and *l* = 4 mm.

## 3. Fabrication of the F-P Sensor

**Figure 5:**
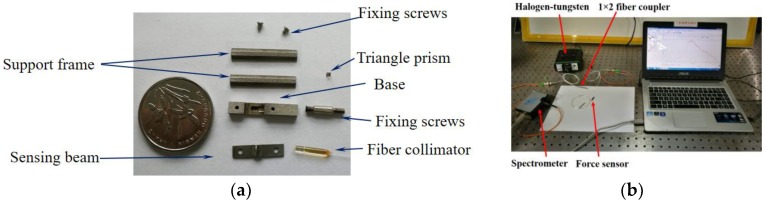

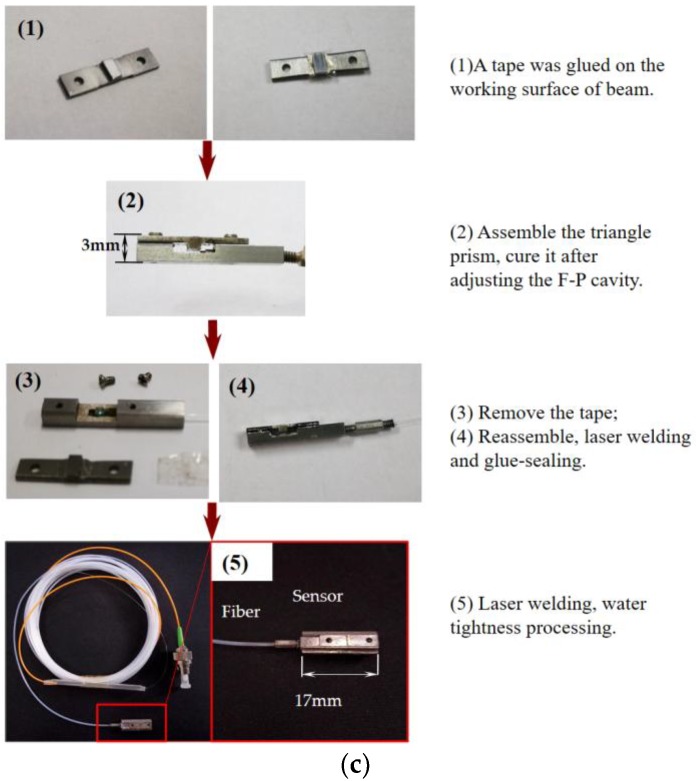
(**a**) All elements of the sensor, (**b**) the sensor signal monitoring system, (**c**) the preparation process of the sensor. There are five key steps in the sensor production.

The F-P force sensor consists of a base, two support frames, a sensing beam, a triangle prism, a fiber collimator, and fixing screws (see Figure 5a). Meanwhile, several preparation steps should be done before the assembly, such as the cleaning of the mechanical structure using the ultrasonic oscillator. In order to ensure the parallelism between the two reflective surfaces of the F-P cavity and the accuracy of the F-P cavity length, the sensor signal monitoring system was used (see Figure 5b). One end of the 1 × 2 fiber coupler was connected to the F-P sensor, and another two ends were connected to a halogen-tungsten light source and a spectrometer, respectively. The spectral signal of the F-P cavity could be obtained by the spectrometer, which can monitor the length of F-P cavity in real-time.

As shown in Figure 5c, there are five key steps to completing the assembly. (1) A 50 μm-thick tape was applied smoothly on the working surface of the force sensing beam to guarantee the length of the F-P cavity. (2) The triangle prism and fiber collimator (with fixing screws) were assembled in the groove of the sensor base. Then, the force sensing beam with 50 μm-thick tape was fixed on the base by two small screws. After that, the triangle prism was adjusted until good F-P cavity signal was obtained. (3) High-temperature glue was used to fix the triangle prism. The force sensing beam was disassembled and the tape was removed. (4) The force sensing beam was reassembled and welded onto the base by a laser welder. (5) Two support frames were welded onto the side edges of the base, and the water tightness processing was done. The overall dimensions of the encapsulated fiber F-P sensor were 17 mm × 5 mm × 3 mm.

## 4. F-P Sensor Experiments

### 4.1. Calibration of F-P Sensor

**Figure 6:**
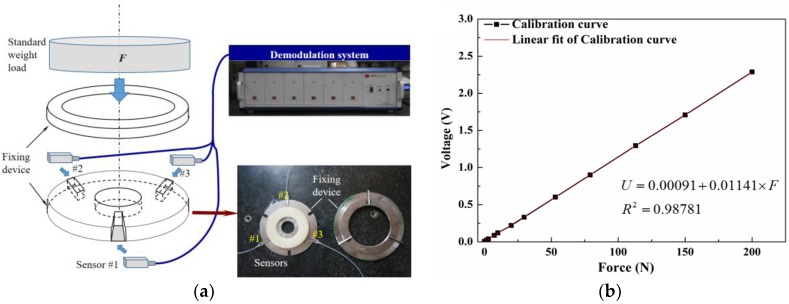
(**a**) Schematic and image of sensors calibration fixture; (**b**) the calibration result and the fitting curve of voltage–force.

A three-point fixing device—a convenient calibration device with a large calibration range and high accuracy—was used to calibrate the fiber F-P force sensor, as shown in Figure 6a. This disc-shaped fixing device has three fixed spaces, all with a 120° angle with a dimensional accuracy of 0.01 mm. Since the output voltage of the CCD varies linearly with its position, a linear fitting was done on the exerted load *F* and the variation of the F-P cavity length to obtain the voltage–force curve. The fitting had a correlation coefficient of 0.98781, and there was a good linearity between the voltage and force. The calibration results and the fitting curve show that the measuring range of the F-P force sensor was 0–200 N with a resolution of 1 N, as shown in Figure 6b. 

### 4.2. Experiments in Humid and High-Pressure Environment

**Figure 7:**
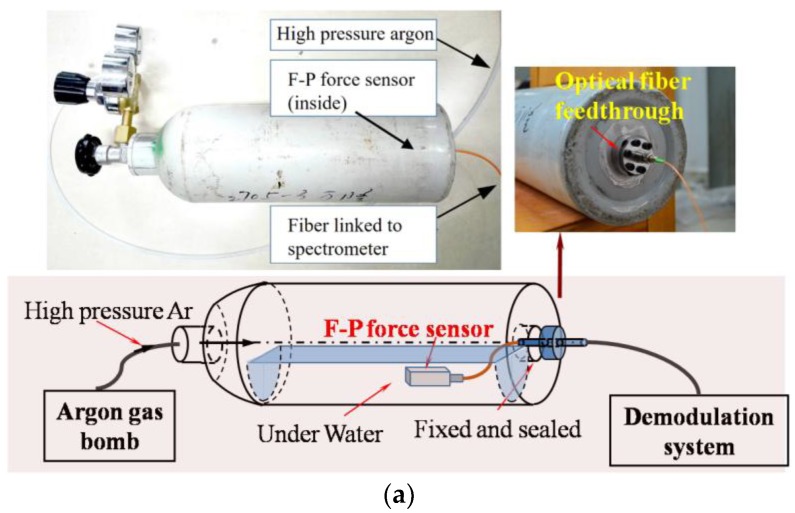

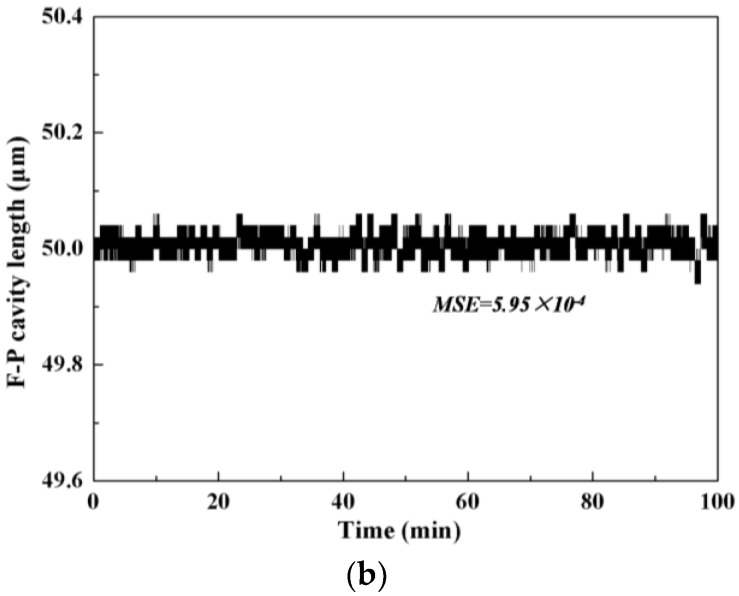
(**a**) The schematic and measurement equipment of the experiment; (**b**) the cavity length results of the sensor in the humid and high-pressure environment. MSE: mean squared error.

A high-pressure gas cylinder injected with argon gas (10 MPa) and water inside was used to simulate the humid and high-pressure environment around the SGT. In order to place a fiber F-P force sensor into the cylinder, an optical fiber feedthrough was installed at the bottom of the cylinder. The F-P force sensor was connected with the optical fiber feedthrough, and the other end of optical fiber feedthrough was connected to the sensor signal monitoring system for a real-time measurement. A detailed schematic and an image of the measurement equipment are shown in Figure 7a. The experimental results in the humid and high-pressure environments are shown in Figure 7b, which indicates that the sensor had a stable response in the rigorous working environments. Mean squared error (MSE) was used to assess the stability of the sensor, and the calculated MSE value of the demodulated F-P cavity of the sensor was 5.95 × 10^−4^.

### 4.3. Experiments in Vibration Environment

**Figure 8:**
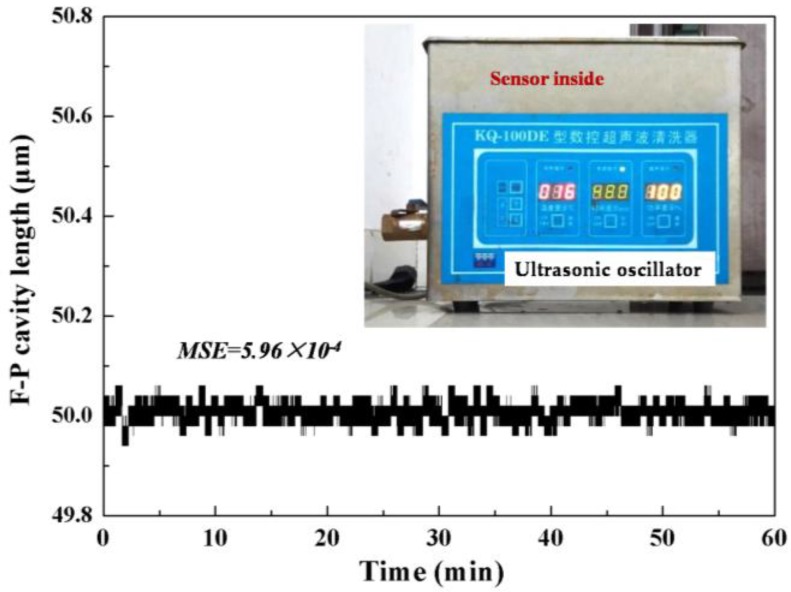
Experimental results of the demodulated F-P cavity values when F-P sensor is in vibration environment.

The vibration of a steam generator tube is caused by the collision of the water flow when the nuclear power plant is running. The vibration strength of the running SGT is not large, so an ultrasonic oscillator was sufficient to simulate the vibration environment in the experiment. The F-P force sensor was ultrasonicated with a vibration frequency of 40 KHz and monitored in real-time by the demodulation system for 60 min. The demodulated F-P cavity length of the force sensor is given in Figure 8, and the corresponding MSE value of the demodulated F-P cavity is 5.96 × 10^−4^, indicating that the vibration environment would not cause degradation of the sensor performance.

### 4.4. Experiments in High-Temperature Environment

**Figure 9:**
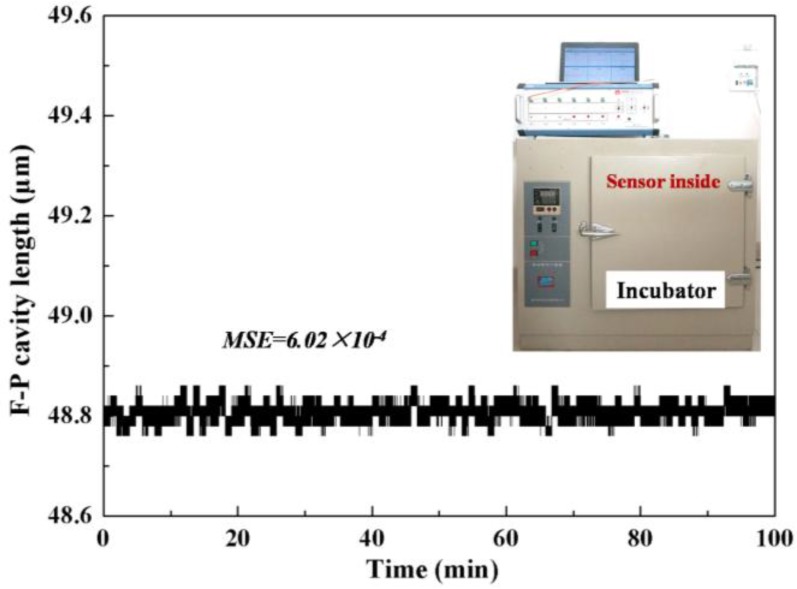
The result of the cavity length of the sensor in the high temperature environment.

In a running PWR nuclear power plant, the average temperature of the primary loop is about 350 °C, and the average temperature of the secondary loop is about 200 °C. Therefore, in order to study the performance of the sensor in the high-temperature environment, the sensor was installed in an incubator (test temperature was set as 350 °C) and its signal was demodulated by the demodulation system in real-time. As shown in Figure 9, the experimental results indicated that the F-P cavity length became smaller (48.8 μm), but the contrast of the sensor signal at 350 °C was the same as at room temperature. Therefore, the small variation of the original F-P cavity length caused by the high temperature did not affect the performance of force sensor. Meanwhile, the calculated MSE value of 6.02 × 10^−4^ indicates that the sensor works well in the high-temperature environment.

### 4.5. Experiments in the 1:1 Steam Generator Test Loop

**Figure 10:**
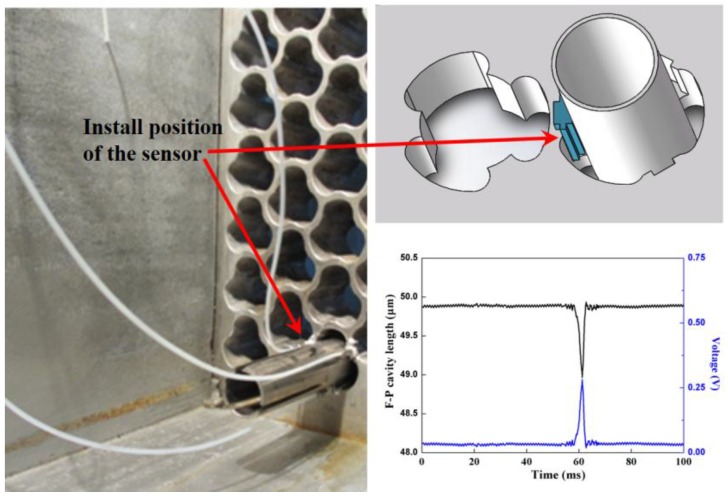
Installed sensors in 1:1 steam generator test loop and the F-P cavity length and the CCD output voltage of one test point.

The fiber F-P sensors were exploited to measure the radial impact force between the SGT and the TSP in a 1:1 steam generator test loop with the cooperation of the nuclear power institute of China. The fiber F-P force sensors were installed in the gap between the SGT and the TSP (see Figure 10). During the test in the 1:1 steam generator test loop, there were 30 F-P sensor test points, and the measuring result of one of the test points is shown in Figure 10. According to the calibration, the radial collision force between the SGT and the TSP was about 22 N.

## 5. Conclusions

We demonstrated a F-P sensor which is capable of measuring the radial collision force of a SGT. The sensor with a dimension of 17 × 5 × 3 mm^3^ showed good performance in humid, high-pressure and vibration environment, with a force measurement range of 0–200 N and a resolution of 1 N. The sensor was suitable for installation in the small contact position between the SGT and the TSP of a real 1:1 steam generator test loop. Next, we will investigate the corresponding optical demodulation system.

## Figures and Tables

**Table 1:** The calculation results when the clamped-clamped beams were used with 06Cr19Ni10 and 3Cr13 in different beam length.

Material	Parameters	*l* (mm)	*S* (nm/N)	*F_m_* (N)	*f*_0_ (Hz)	Accuracy (N)	Maximal Force (N)
06Cr19Ni10	*E* = 193 GPa	3	20.24	166.4	13,959	1.48	1235.20
	*σ_b_* = 520 MPa	4	47.98	124.8	7852.0	0.63	521.10
	*ρ* = 7.75 g/cm^3^	5	93.70	99.8	5025.3	0.32	266.80
3Cr13	*E* = 216.5 GPa	3	18.04	272	14,784	1.66	1385.6
	*σ_b_* = 850 MPa	4	42.77	204	8316.2	0.70	584.55
	*ρ* = 7.93 g/cm^3^	5	83.53	163.2	5322.4	0.36	299.29
